# Real-World Outcomes of First-Line Pertuzumab, Trastuzumab, and Taxane in HER2-Positive Metastatic Breast Cancer in Costa Rica: A Multicenter Retrospective Study

**DOI:** 10.7759/cureus.104856

**Published:** 2026-03-08

**Authors:** Khanna Priyanka, Denis U Landaverde

**Affiliations:** 1 Medical Oncology Department, Hospital Dr. Maximiliano Peralta Jiménez, Caja Costarricense de Seguro Social, Cartago, CRI; 2 Medical Oncology Department, Hospital México, Caja Costarricense de Seguro Social, San Jose, CRI

**Keywords:** costa rica, effectiveness and safety, her2-positive metastatic breast cancer, real-world clinical practice, treatment of the breast cancer

## Abstract

Background

Dual HER2 blockade with pertuzumab, trastuzumab, and a taxane is the current standard first-line treatment for HER2-positive metastatic breast cancer (mBC), based on pivotal trials such as the CLEOPATRA. Since 2014, this regimen has been implemented in the Costa Rican public healthcare system. However, real-world data from Central America are lacking.

Methods

We conducted a retrospective, multicenter observational study of 148 patients with histologically confirmed HER2-positive mBC who were treated between August 2015 and August 2021 at five Costa Rican public hospitals. Primary endpoints were progression-free survival (PFS), overall survival (OS), and safety profile. Survival analysis was performed using Kaplan-Meier estimates.

Results

Median age was 58 years; 95% had ECOG 0-1; 54% were hormone receptor-positive. Visceral metastases were present in 37.8%, and 4.7% had brain metastases at diagnosis. Paclitaxel was the most used taxane (85%). Median duration of anti-HER2 therapy was 22.7 months. Median PFS was 19 months (95% CI: 15-25), and median OS was 73 months (95% CI: 38-74), with a median follow-up of 27.5 months. Most adverse events were grade 1-2, with peripheral sensory neuropathy (34%) and diarrhea (21%) being the most common. Grade 3 cardiotoxicity occurred in two patients. Subgroup analysis by hormone receptor status showed no statistically significant differences in PFS (HR-positive: 18.2 months (95% CI 14.1-22.3) vs. HR-negative: 20.1 months (95% CI 15.8-24.4); p = 0.42) or OS (HR-positive: 71 months (95% CI 36-106) vs. HR-negative: 74 months (95% CI 40-108); p = 0.38).

Conclusion

This first Central American real-world study demonstrates that first-line pertuzumab, trastuzumab, and taxane yields PFS, OS, and safety outcomes comparable to pivotal trials in HER2-positive mBC, despite including patients with prior trastuzumab exposure and brain metastases. These findings contribute to regional real-world evidence and underscore the need for neurological monitoring, given the high rate of central nervous system (CNS) progression. Cross-study comparisons remain descriptive due to population and design differences.

## Introduction

Breast cancer is the most frequently diagnosed malignancy worldwide, with approximately 2.3 million new cases in 2020 [1]. In Costa Rica, it represents the leading cause of cancer-related mortality among women and has the highest incidence in Central America [1,2]. HER2-positive breast cancer accounts for 15%-20% of cases and has historically been associated with aggressive behavior [3,4]. However, HER2-targeted therapies have significantly modified its natural history [5,6].

The CLEOPATRA trial established the combination of pertuzumab, trastuzumab, and docetaxel as the standard first-line therapy for HER2-positive metastatic breast cancer (mBC), demonstrating significant improvements in progression-free (PFS) and overall survival (OS) [7,8]. Subsequent studies, including PERUSE, confirmed the benefit of dual HER2 blockade combined with various taxanes in broader patient populations [9,10].

Although randomized controlled trials provide the highest level of evidence, their strict criteria often limit external validity. Real-world evidence is essential to determine whether clinical trial benefits are reproducible in heterogeneous populations treated in routine practice [11].

This first Central American real-world study evaluates PFS, OS, and toxicity of first-line pertuzumab, trastuzumab, and taxane in HER2-positive mBC within the Costa Rican public healthcare system.

## Materials and methods

Study design and setting

This was a retrospective, multicenter, observational cohort study conducted across five tertiary referral hospitals within the Costa Rican public healthcare system (Caja Costarricense de Seguro Social, CCSS): Hospital México, Hospital Calderón Guardia, Hospital San Juan de Dios, Hospital San Vicente de Paul, and Hospital Max Peralta.

The study evaluated real-world outcomes of patients treated between August 2015 and August 2021. The research protocol was reviewed and approved by the Centro de Desarrollo Estratégico e Información en Salud y Seguridad Social (CENDEISSS) Subárea de Bioética en Investigación, Caja Costarricense de Seguro Social, under CCSS protocol number R022-SABI-00310. Given the retrospective design and use of anonymized data, informed consent was waived.

Study population

Eligible patients met the following inclusion criteria: age ≥ 18 years, histologically confirmed HER2-positive mBC, no prior systemic therapy for metastatic disease, and receipt of first-line therapy with pertuzumab, trastuzumab, and a taxane (paclitaxel or docetaxel).

HER2 positivity was determined according to institutional standards based on immunohistochemistry and/or in situ hybridization, consistent with contemporary clinical practice guidelines during the study period. Notably, while 67.1% of patients had received adjuvant trastuzumab for early-stage disease, no patients had received pertuzumab in the neoadjuvant or adjuvant setting, as pertuzumab was not approved for early-stage breast cancer in Costa Rica during the study period.

Due to the observational nature of the study, no exclusion criteria were predefined to reflect routine clinical practice and preserve external validity.

Data collection

Variables extracted included demographic characteristics (age, province of origin); clinical characteristics (ECOG performance status, stage at diagnosis, and prior therapies); tumor biology (histology and hormone receptor status); sites of metastatic disease; treatment exposure (type of taxane, number of cycles, and duration of anti-HER2 therapy); reasons for treatment discontinuation; and dates of progression and death.

Study endpoints

Primary Endpoints

PFS was defined as the time from initiation of first-line therapy to the first documented event among the following: radiologic progression per RECIST criteria (where available), clinical progression documented by the treating physician leading to treatment modification, treatment discontinuation due to toxicity (grade ≥3), or death from any cause.

Disease progression was determined by the treating physician based on clinical assessment and/or radiologic imaging (CT, PET/CT, or bone scan) using RECIST criteria where available. Due to the retrospective real-world design, exact radiographic response per RECIST was not uniformly documented across all centers; therefore, PFS was determined based on medical record documentation of progression events leading to treatment change.

Patients were followed according to institutional practice, typically with clinical evaluation every 3-4 weeks during taxane administration and every 3 months thereafter. Radiologic assessments were performed at baseline and subsequently at physician's discretion or when clinically indicated, generally every 3-6 months. Treatment delays of less than 4 weeks did not constitute treatment discontinuation; patients who resumed therapy after brief interruptions remained in the analysis. Only permanent discontinuation (due to progression, toxicity, death, or patient decision) was considered as a treatment endpoint.

OS was defined as the time from initiation of first-line therapy to death from any cause or last follow-up.

Secondary Endpoints

Secondary endpoints included safety profile according to the Common Terminology Criteria for Adverse Events (CTCAE), treatment exposure duration, and reasons for treatment discontinuation. Reported toxicities were recorded and graded according to CTCAE criteria as documented in the medical records.

Statistical analysis

Descriptive statistics were used to summarize demographic, clinical, and pathological characteristics. Categorical variables were expressed as frequencies and percentages. Continuous variables were reported as means with standard deviations or medians when appropriate.

Survival curves were estimated using the Kaplan-Meier method. Comparisons between groups were performed using the log-rank test. A Cox proportional hazards model was applied to evaluate the impact of clinical variables on progression-free and overall survival.

All statistical analyses were performed using IBM SPSS Statistics for Windows, Version 20.0 (Released 2011; IBM Corp., Armonk, NY, USA). A two-sided p-value < 0.05 was considered statistically significant.

## Results

**Figure 1:**
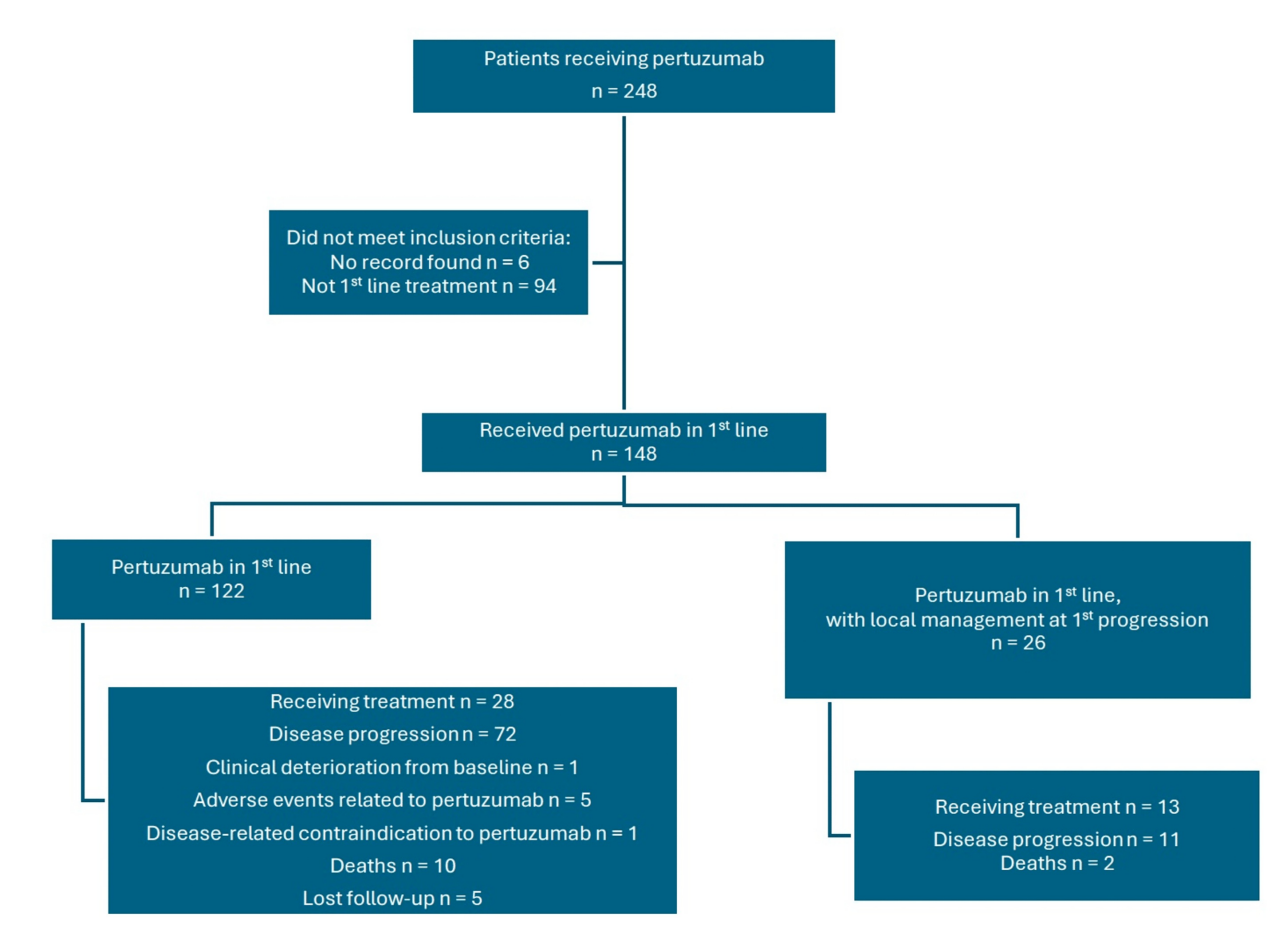
CONSORT diagram of the patient selection process

Between August 2015 and August 2021, a total of 248 patients received pertuzumab during the study period. Of them, 148 patients met eligibility criteria and received pertuzumab in combination with trastuzumab and a taxane as first-line therapy for metastatic disease (Figure 1).

Baseline demographic and clinicopathologic characteristics are summarized in Table 1. The median age at initiation of first-line therapy was 58 years. Most patients (95%) had a good performance status (ECOG 0-1). Hormone receptor positivity was documented in 54% of cases. At initial breast cancer diagnosis, 52.7% had stage IV disease, while the remainder had prior early or locally advanced disease.

**Table 1:** Baseline demographics and clinicopathologic characteristics

Variable	N = 148 (%)
Age (years)	
<40	16 (10.8)
40-49	24 (16.2)
50-59	44 (29.7)
60-69	47 (31.8)
≥70	17 (11.5)
Year of diagnosis	
<2009	7 (4.7)
2010-2014	28 (18.9)
2015-2019	79 (53.4)
≥2020	33 (22.3)
Stage at diagnosis	
I	7 (4.7)
II	25 (16.9)
III	37 (25.0)
IV	78 (52.7)
Unknown	1 (0.7)
Histological type	
Ductal	145 (98.0)
Lobular	3 (2.0)
Hormonal receptors	
Negative	67 (45.3)
Positive	81 (54.7)

Regarding prior treatments in early-stage disease, 90% of patients had received neoadjuvant or adjuvant chemotherapy, and 67% had been exposed to trastuzumab in the adjuvant setting (Table 2). Notably, while 67.1% of patients had received adjuvant trastuzumab for early-stage disease, no patients had received pertuzumab in the neoadjuvant or adjuvant setting, as pertuzumab was not approved for early-stage breast cancer in Costa Rica during the study period.

**Table 2:** Management of patients with early-stage or locally advanced breast cancer

Variable	N = 70 (%)
Neoadjuvant/adjuvant chemotherapy	
No	4 (5.7)
Yes	65 (92.9)
Unknown	1 (1.4)
Trastuzumab adjuvant treatment	
No	20 (28.6)
Yes	47 (67.1)
Not required	2 (2.9)
Unknown	1 (1.4)
Adjuvant hormonal therapy	
No	8 (11.4)
Yes	46 (65.7)
Not required	15 (21.4)
Unknown	1 (1.4)

At the time of metastatic presentation, most patients maintained a favorable functional status (Table 3). The predominant pattern of metastatic involvement was non-visceral disease (71.6%), while 37.8% had visceral metastases and 4.7% had documented brain metastases at baseline (Table 4).

**Table 3:** Performance status of patients in metastatic disease ECOG: Eastern Cooperative Oncology Group performance status scale.

Variable	N = 148 (%)
ECOG	
0	78 (52.7)
1	61 (41.2)
2	7 (4.7)
Unknown	2 (1.4)

**Table 4:** Pattern of metastatic disease

Metastasis	N = 148 (%)
Brain	7 (4.7)
Visceral	56 (37.8)
Non-visceral	106 (71.6)

Treatment exposure and discontinuation

Paclitaxel was the most frequently administered taxane (85%), followed by docetaxel (14%), as detailed in Table 5. Patients receiving paclitaxel completed a mean of 16 weekly cycles (Table 6). The median duration of dual anti-HER2 therapy (pertuzumab plus trastuzumab) was 22.7 months.

**Table 5:** Cytotoxic agents used in first-line treatment and reasons for discontinuation

Variable	N = 148 (%)
Body surface area (mean ± SD)	1.67 ± 0.17
Taxane used	
Paclitaxel	126 (85.1)
Docetaxel	21 (14)
Unknown	1 (0.7)
Discontinuation	
Baseline deterioration	1 (0.7)
Toxicity	5 (3.4)
Discontinued therapy voluntarily	5 (3.4)
Death	12 (8.1)
Receiving treatment	42 (28.4)
Disease progression	83 (56.1)

**Table 6:** Number of cycles and dose of taxanes received in metastatic disease

Chemotherapy	Mean	SD	Minimum	Maximum
No. of cycles				
Paclitaxel	16.2	6.2	1	40
Docetaxel	10.5	8.6	2	40
Dose received (mg)				
Paclitaxel	136.0	26.1	100	294
Docetaxel	129.7	12.2	108	150

At data cutoff, the most common reason for treatment discontinuation was disease progression (56.1%), followed by death (8%). Treatment discontinuation due to toxicity occurred in 3.4% of patients, and 3.4% discontinued therapy voluntarily (Table 5).

Among patients with disease progression, the central nervous system (CNS) was the most frequent site of first documented progression (38.5%).

Subgroup analysis by prior trastuzumab exposure

Among the 47 patients (67.1% of those with early-stage disease) who had received prior adjuvant trastuzumab, median PFS was 17.2 months (95% CI 13.4-21.0) and median OS was 68 months (95% CI 34-102). These outcomes demonstrate that dual HER2 blockade remains effective even in patients with prior trastuzumab exposure, a population that represents an increasingly common real-world scenario and was largely excluded from the CLEOPATRA trial, which required a ≥12-month treatment-free interval from adjuvant therapy.

Subgroup analysis by hormone receptor status

Subgroup analysis by hormone receptor status showed no statistically significant difference in PFS (HR-positive: median 18.2 months (95% CI 14.1-22.3) vs. HR-negative: median 20.1 months (95% CI 15.8-24.4); log-rank p = 0.42) or OS (HR-positive: median 71 months (95% CI 36-106) vs. HR-negative: median 74 months (95% CI 40-108); p = 0.38).

Safety profile

Treatment-related adverse events are summarized in Table 7. Most toxicities were grade 1-2 according to CTCAE criteria. The most frequently reported adverse events were peripheral sensory neuropathy (34%), diarrhea (21%), rash (12%), and nausea (3%). Febrile neutropenia occurred in 1% of patients. Grade 3 cardiotoxicity was observed in two patients (1.3%). Overall, severe toxicity leading to treatment discontinuation was uncommon.

**Table 7:** Treatment-related adverse events

Adverse events	N = 148 (%)	Grade, N (%)				
		1	2	3	4	Unknown
Diarrhea	31 (21)	24 (77)	5 (16)	1 (3)	0	1 (3)
Vomiting	2 (1)	0	2 (100)	0	0	0
Nausea	5 (3)	4 (80)	1 (20)	0	0	0
Rash	18 (12)	12 (67)	4 (22)	0	0	2 (11)
Neutropenia	2 (1)	0	0	1 (50)	1 (50)	0
Peripheral sensory neuropathy	50 (34)	24 (48)	17 (34)	6 (12)	0	3 (6)
Cardiotoxicity	11 (7)	4 (36)	5 (45)	2 (18)	0	0

Survival outcomes

**Figure 2:**
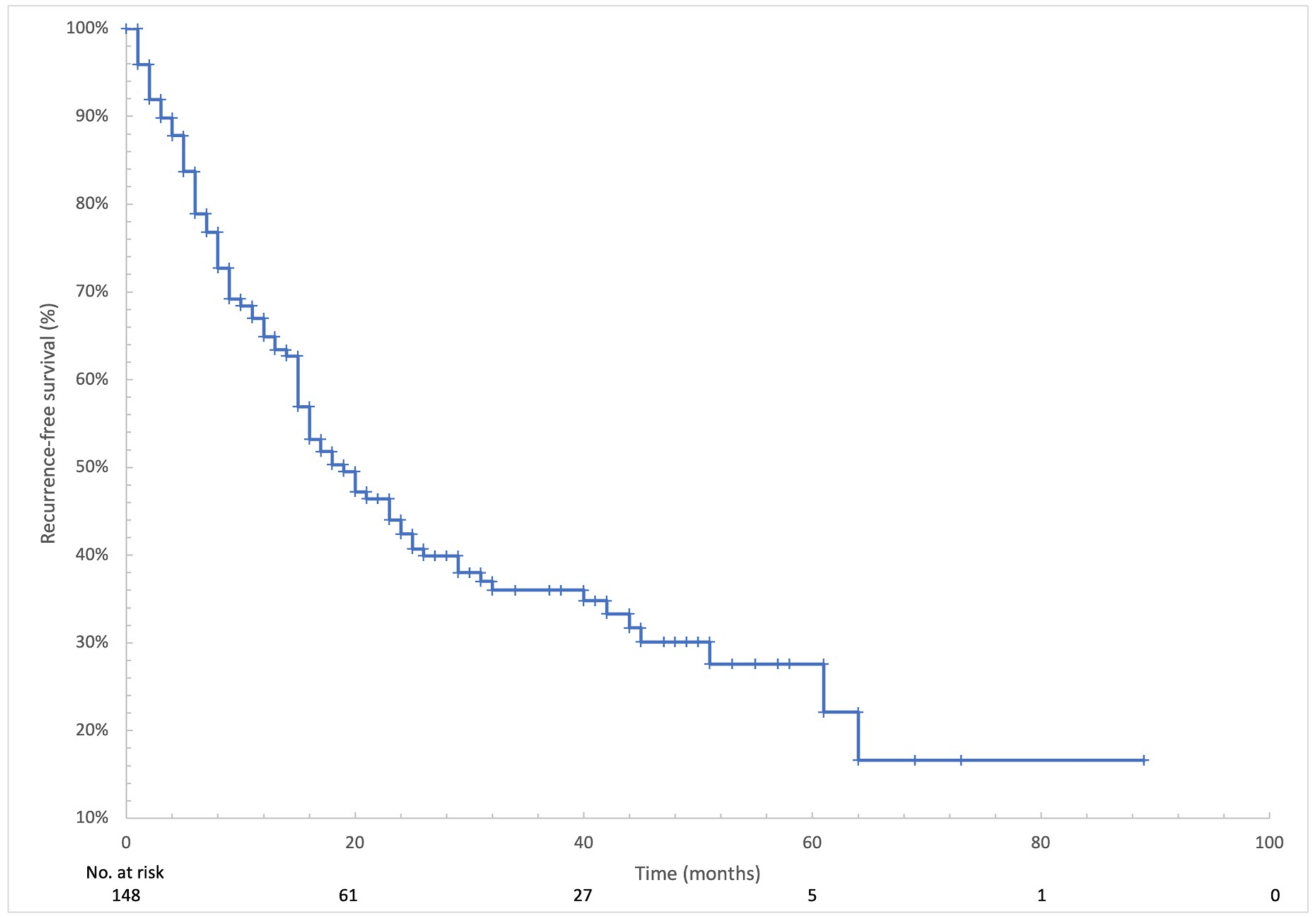
Kaplan-Meier curve of recurrence-free survival

**Figure 3:**
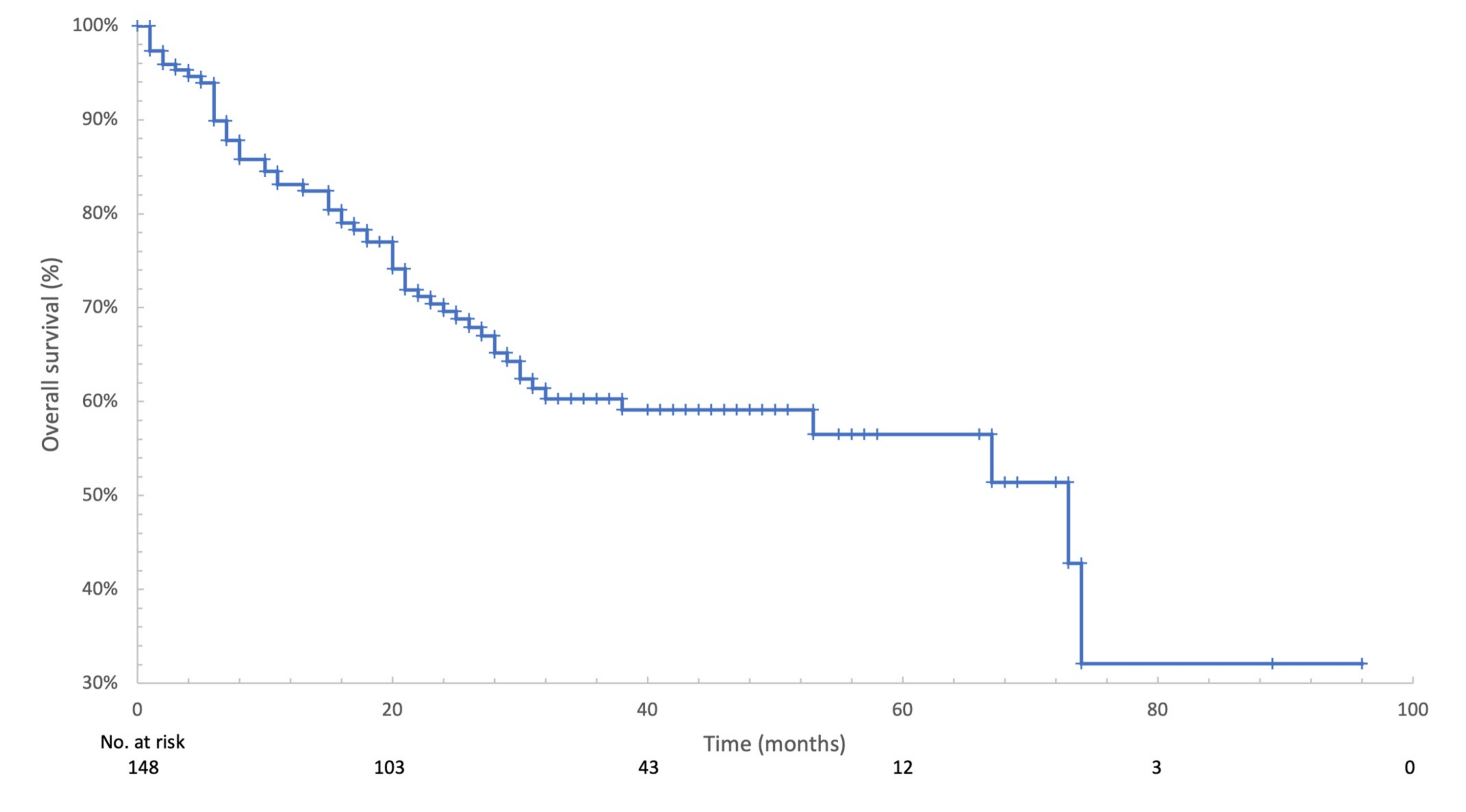
Kaplan-Meier curve of overall survival

After a median follow-up of 27.5 months, median PFS was 19 months (95% CI: 15-25), as shown in Figure 2. Median OS was 73 months (95% CI: 38-74), as illustrated in Figure 3. Subgroup analyses did not demonstrate statistically significant differences in PFS or OS according to site of metastasis, ECOG performance status, or taxane used.

## Discussion

This multicenter real-world study demonstrates that first-line pertuzumab, trastuzumab, and a taxane provide sustained clinical benefit in patients with HER2-positive mBC treated within the Costa Rican public healthcare system. Our findings suggest that outcomes reported in pivotal clinical trials are reproducible in routine practice.

The baseline clinical characteristics of our cohort were similar to those reported in CLEOPATRA, with most patients younger than 65 years, good performance status, and a substantial proportion of hormone receptor-positive tumors [8,10]. However, important differences were observed.

A significantly higher proportion of our patients had received prior neoadjuvant or adjuvant therapy, including trastuzumab exposure (69% vs. 10% in CLEOPATRA). Prior anti-HER2 therapy could potentially influence resistance mechanisms and disease biology [5,6]. Despite this, survival outcomes remained comparable to those reported in CLEOPATRA and PERUSE, reinforcing the robustness of dual HER2 blockade in real-world populations.

Our cohort also included patients with brain metastases, unlike CLEOPATRA. Additionally, we observed a lower proportion of visceral metastatic disease (37%) compared with PERUSE (69%) and CLEOPATRA (78%). These differences reflect the heterogeneity of real-life practice and highlight why direct numerical comparisons between trial and observational cohorts must be interpreted cautiously.

Paclitaxel was the most frequently used taxane, consistent with evolving practice patterns following PERUSE, particularly due to its tolerability profile [7]. Patients in our cohort received a mean of 16 weekly paclitaxel administrations, exceeding the six cycles reported in PERUSE [9,10] and differing from the docetaxel backbone used in CLEOPATRA.

The median duration of dual anti-HER2 therapy was 22.7 months (approximately 29 applications), comparable to treatment exposure reported in PERUSE and CLEOPATRA. As in pivotal studies, disease progression remained the main reason for treatment discontinuation.

Our findings align with those reported in pivotal trials. Median PFS (19 months) was comparable to PERUSE (20.7 months) and CLEOPATRA (18.7 months) [8,9]. Median OS (73 months) exceeded both PERUSE (65.3 months) and CLEOPATRA (57.1 months), though cross-trial comparisons should be interpreted cautiously given differences in population characteristics, including higher rates of prior trastuzumab exposure in our cohort and the inclusion of patients with brain metastases [9,10].

The median OS of 73 months exceeding that reported in pivotal trials warrants consideration of contributing factors. While first-line dual HER2 blockade undoubtedly contributed to this outcome, the availability of effective subsequent therapies likely played a substantial role. During the study period, T-DM1 was available for eligible patients as second-line therapy based on the EMILIA trial results, and lapatinib-based regimens or capecitabine were also accessible [12]. This sequential anti-HER2 strategy dual blockade followed by T-DM1 or other HER2-directed therapies reflects modern treatment paradigms and likely contributed to prolonged survival. However, we acknowledge that complete second-line treatment data were not systematically collected for all patients, which represents a limitation of this study.

In PERUSE, multivariate analysis suggested that visceral disease and prior trastuzumab exposure were associated with inferior outcomes [9,10]. In contrast, our subgroup analysis did not demonstrate statistically significant differences, possibly due to sample size limitations or differences in disease distribution.

Given that 54.7% of our population was hormone receptor-positive and the CNS was the most frequent site of first progression (38.5%), optimizing maintenance strategies for HR+/HER2+ mBC remains an important clinical priority. The PATINA trial (NCT02947685) is evaluating the addition of palbociclib to pertuzumab, trastuzumab, and endocrine therapy versus trastuzumab and endocrine therapy alone after induction chemotherapy. While our cohort did not receive CDK4/6 inhibitors, the high rate of CNS progression observed highlights the need for continued investigation into maintenance approaches that may offer both systemic and intracranial disease control. Future studies incorporating CDK4/6 inhibition with dual HER2 blockade and endocrine therapy will help clarify whether such strategies can reduce the burden of CNS progression in this population.

The safety profile observed was consistent with previous reports. Most adverse events were grade 1-2. The most common toxicities were diarrhea, rash, and peripheral neuropathy. Severe cardiotoxicity and febrile neutropenia were infrequent but clinically relevant, as previously described. Treatment discontinuation due to toxicity was uncommon, supporting the tolerability of this regimen in routine practice.

Brain metastases were the most frequent site of progression, consistent with known patterns of HER2-positive mBC [3,4]. This observation reinforces the importance of careful neurologic monitoring in patients receiving dual HER2 blockade and highlights the CNS as a persistent site of vulnerability in HER2-positive metastatic disease.

Limitations

This study is limited by its retrospective design. Missing data, potential selection bias, and heterogeneity in radiologic response documentation are inherent to real-world analyses. Additionally, differences in follow-up intensity and imaging availability across centers may have influenced outcome assessment.

Although our median follow-up of 27.5 months is adequate for PFS assessment, OS data should be interpreted with consideration of follow-up maturity. With only 27.5 months median follow-up and an estimated median OS of 73 months, a substantial proportion of patients were censored alive, which may introduce uncertainty in long-term survival estimates. Furthermore, subgroup analyses were limited by statistical power; with 148 patients, we had approximately 80% power to detect hazard ratios of 0.60 or lower in subgroups comprising at least 30% of the population, but smaller subgroups may be underpowered to detect clinically meaningful differences. These limitations reflect the inherent trade-offs of real-world retrospective research and underscore the need for larger, prospective regional registries.

Nevertheless, the inclusion of patients treated in five tertiary hospitals strengthens the external validity of our findings and provides representative data from a national public healthcare system.

Clinical implications

Our findings have several clinical implications. First, they suggest that dual HER2 blockade with pertuzumab and trastuzumab remains effective in patients previously exposed to adjuvant trastuzumab, a scenario increasingly common in modern oncology practice. Second, the high rate of CNS progression underscores the need for systematic neurologic evaluation and potentially earlier imaging in symptomatic patients. Third, the tolerability profile observed in routine practice supports the use of weekly paclitaxel as a feasible chemotherapy backbone in older or comorbid patients.

Expanding real-world evidence generation in Latin America is critical to guide clinical practice, improve equity in access to innovative therapies, and strengthen regional oncology research infrastructure.

## Conclusions

This multicenter real-world study demonstrates that first-line pertuzumab, trastuzumab, and a taxane provide outcomes comparable to those reported in pivotal clinical trials for patients with HER2-positive mBC treated within the Costa Rican public healthcare system, despite including patients with prior trastuzumab exposure and brain metastases. The safety profile was consistent with previous reports. These findings contribute real-world evidence from a Central American population and highlight the importance of systematic neurological monitoring, given the observed rate of CNS progression. Cross-study comparisons should be interpreted descriptively, given differences in population characteristics and study design.
